# Chronic kidney disease and support provided by home care services: a systematic review

**DOI:** 10.1186/1471-2369-15-118

**Published:** 2014-07-18

**Authors:** Sema K Aydede, Paul Komenda, Ognjenka Djurdjev, Adeera Levin

**Affiliations:** 1 School of Population and Public Health, The University of British Columbia and Provincial Health Services Authority, 700-1380 Burrard Street, Vancouver, BC V6Z 2H3, Canada; 2 Faculty of Medicine, Section of Nephrology, University of Manitoba and Seven Oaks General Hospital, Room 2PD02 – 2300 McPhillips Street, Winnipeg, MB R2V 3M3, Canada; 3 British Columbia Provincial Renal Agency, Providence Bldg, Room 570.4, 1081 Burrard Street, Vancouver, BC V6Z 1Y6, Canada; 4 Division of Nephrology, Providence Bldg, Room 6010A, The University of British Columbia and British Columbia Provincial Renal Agency, 1081 Burrard Street, Vancouver, BC V6Z 1Y6, Canada

**Keywords:** Chronic kidney disease, Dialysis, Home care services

## Abstract

**Background:**

Chronic diseases, such as chronic kidney disease (CKD), are growing in incidence and prevalence, in part due to an aging population. Support provided through home care services may be useful in attaining a more efficient and higher quality care for CKD patients.

**Methods:**

A systematic review was performed to identify studies examining home care interventions among adult CKD patients incorporating all outcomes. Studies examining home care services as an alternative to acute, post-acute or hospice care and those for long-term maintenance in patients’ homes were included. Studies with only a home training intervention and those without an applied research component were excluded.

**Results:**

Seventeen studies (10 cohort, 4 non-comparative, 2 cross-sectional, 1 randomized) examined the support provided by home care services in 15,058 CKD patients. Fourteen studies included peritoneal dialysis (PD), two incorporated hemodialysis (HD) and one included both PD and HD patients in their treatment groups. Sixteen studies focused on the dialysis phase of care in their study samples and one study included information from both the dialysis and pre-dialysis phases of care. Study settings included nine single hospital/dialysis centers and three regional/metropolitan areas and five were at the national level. Studies primarily focused on nurse assisted home care patients and mostly examined PD related clinical outcomes. In PD studies with comparators, peritonitis risks and technique survival rates were similar across home care assisted patients and comparators. The risk of mortality, however, was higher for home care assisted PD patients. While most studies adjusted for age and comorbidities, information about multidimensional prognostic indices that take into account physical, psychological, cognitive, functional and social factors among CKD patients was not easily available.

**Conclusions:**

Most studies focused on nurse assisted home care patients on dialysis. The majority were single site studies incorporating small patient populations. There are gaps in the literature regarding the utility of providing home care to CKD patients and the impact this has on healthcare resources.

## Background

The world population is aging and the segment of global population 60 years of age and over is increasing at the fastest pace ever seen in history [1]. The population trends are reflected in the demographic profiles of patients with diseases such as chronic kidney disease (CKD) that are common in the elderly. In Canada, over half of the patients initiating renal replacement therapy (RRT) in 2009 were 65 years of age and older [2]. In Europe, RRT patients 65 years of age and older had the highest rate of increase in prevalence over the 1992–2005 period [3].

CKD populations, especially elderly end-stage renal disease (ESRD) patients, are faced with multiple medical and social challenges such as having to cope with several comorbidities, physical disability, cognitive impairment and social isolation [4-11]. These medical and social challenges are significant in characterizing the impaired quality of life in CKD patients [12,13]. Quality of life deteriorates as the severity of CKD increases [14]. Home care (HC) services may help CKD patients in coping with these challenges, maintaining their independence and fulfilling their preferences of receiving care at home [15,16].

Current emphasis on active aging and independence represent a unique opportunity to examine HC services that are utilized to varying degrees by different patient groups but are considered beneficial especially in chronic conditions [17]. In the case of non-ESRD CKD, the utilization of HC may vary based on patient’s age and comorbidities and, in the case of ESRD, it may vary based on the severity of illness and therapy type. HC services may help in supporting ESRD patients who have chosen conservative care. The independent treatment modalities for ESRD (peritoneal dialysis, PD, and home hemodialysis, HHD), emphasized as viable alternatives to facility-based treatment modalities over the last decade, are less costly to direct service providers, with equivalent or superior patient outcomes and quality of life [18-21]. Patients with ESRD, who are on PD or HHD, however, may utilize more HC services compared to those who are on a facility-based hemodialysis (HD), partially offsetting cost saving [22,23]. On the other hand, the intensity of HC services received may reduce the number of hospitalizations and subsequent health system costs [24-26] regardless of the stage of CKD and the type of therapy for ESRD.

In general, HC services provide support to patients and help them with the daily management of their diseases in their communities. However, a proper characterization and a systematic evaluation of these services within a high risk, resource intense group of patients such as those with CKD have not been undertaken. This systematic review (SR) provides a rigorous account of research evidence on HC use among those with CKD.

## Methods

### Eligibility criteria

Studies about adult patients with any CKD severity level and a HC intervention regarding services provided in patients’ homes were eligible. Our SR was not restricted to studies that incorporated a comparison group. Studies with interventions related to the market place and working conditions of HC professionals or organizational underpinnings of HC organizations were excluded.

In the case of RRT, assisted PD patients could get help from a family member, a friend or a HC worker. In this SR, studies that focused on assisted PD in general without providing subgroup results for HC assisted PD patients [27-32] and those that examined home visits for reasons other than direct HC provision [33-36] were excluded. Unlike assisted PD, HHD is rarely available in an assisted format. One recent study that focused on the feasibility of nocturnal assisted HHD did not provide subgroup results for HC assisted HHD patients and, therefore, was not included in our SR [37].

In the case of palliative care, support services for CKD patients could be provided in their home or at a hospice. This study focused on home-based end-of-life care. Studies that examined palliative care without providing information about the specific services patients received in their community and those that did not separately report on subgroups of patients who received home-based support services [38-42] were excluded from our SR.

The primary outcomes included hospitalizations, admissions to institutional settings, length of stay on independent dialysis modalities for ESRD patients, and outcomes specific to treatment type. As secondary outcomes, mortality, medication management, patient satisfaction, caregiver satisfaction, physical and psychological well-being, health status and quality of life were considered. Studies were not excluded based on outcomes studied.

In general, all types of studies including experimental and observational studies were included. The studies that did not contain an empirical component and those with only training/educational and referral/recommendations types of interventions were excluded.

Given the diversity of HC services and resource considerations, we concentrated on studies published in English. To balance this limitation, a comprehensive literature search was undertaken. The date range was 1990 (i.e., the early stages of profound changes in healthcare systems that started with shifts away from acute care settings towards home and community care [43]) to present.

### Definitions

CKD was conceptualized as consisting of five stages following the Kidney Disease Outcomes Quality Initiative’s definition accepted during the Kidney Disease: Improving Global Outcomes (KDIGO) conference [44]. The definition accepted during the KDIGO conference suggests that CKD could also be classified by treatment type: kidney transplant recipient, CKD independent of dialysis and CKD on dialysis. These classifications guided the development phase of our SR.

The Federal/Provincial/Territorial Working Group on Home Care’s definition, as reported by Health Canada, emphasizes the manner with which HC helps patients: “An array of services which enables clients, incapacitated in whole or part, to live at home, often with the affect of preventing, delaying or substituting for long-term or acute care alternatives” [45]. The Canadian Home Care Association’s definition focuses on the breadth of services covered: “an array of services, provided in the home and community setting, that encompasses health promotion and teaching, curative intervention, end-of-life care, rehabilitation, support and maintenance, social adaptation and integration and support for the family caregiver” [46]. Initially, HC conceptualization for this SR was guided by these definitions. These conceptualizations were further refined during the course of this study as we attempted to standardize terminology for our SR based on HC services covered in the included studies.

### Information sources

Studies were identified through electronic databases, web sites, hand searches and consultations with experts in the field. Electronic databases included MEDLINE, EMBASE, CINAHL, PsycINFO, EconLit, Cochrane CENTRAL, Cochrane Methodology Register, Cochrane Database of Systematic Reviews, Centre for Reviews and Dissemination (DARE, HTA and NHS EED), ACP Journal Club and Web of Science (final search for MEDLINE is incorporated in the Appendix; final searches for other databases are available from the corresponding author). The web sites included World Health Organization, Health Canada, Canadian Homecare Association, Canadian Health Research Collection and the health departments of Canadian provinces and territories. The electronic database and web site searches, conducted by a University of British Columbia librarian, were completed on May 24, 2012. Hand searches, completed on September 20, 2013, were coupled with consultations with experts in the field.

### Study selection

We followed a layered approach in study selection. An initial elimination of irrelevant studies was carried out by study assistants independently based on title and abstract reviews using a pre-tested selection form. An author (SKA) checked the initial selection and reviewed the complete texts of potentially relevant studies. The remaining manuscripts were reviewed in full by two additional authors independently (AL and PK). Disagreements were resolved after discussions among authors. Each selected study was summarized using a pre-tested data extraction form and was evaluated using the Agency for Healthcare Research and Quality risk of bias and confounding form developed for observational studies [47].

### Analysis

Due to the heterogeneity of patient populations considered, interventions examined and health outcomes reported in the studies included in our SR, a meta-analysis was not possible. We performed a narrative summary of studies focusing mainly on key outcomes of importance to the CKD community.

## Results

### Study selection

**Figure 1:**
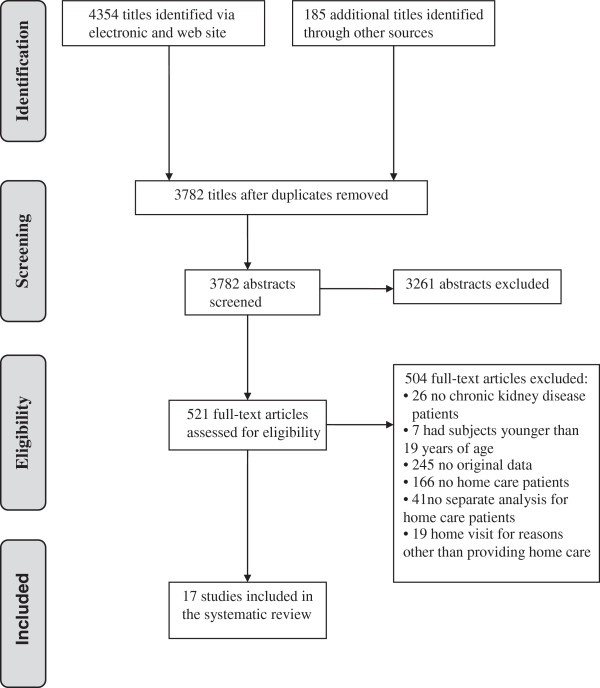
Chronic kidney disease & home care systematic review PRISMA flow diagram.

A total of 17 studies were identified for inclusion in the SR. The searches of electronic databases provided a total of 4354 citations (Figure 1) with 185 additional citations identified through hand-searches and author consultations. After adjustments for duplicates and title and abstract screening, a total of 521 full text articles were assessed and 17 were eligible for inclusion.

### Study characteristics and patient populations

Of the 17 studies included in this study (overview of study characteristics in Table 1 and detail on patient populations, HC interventions and outcomes in Table 2), 14 focused on the impact HC has on PD patients. Ten of the PD studies were cohort studies [48-57], three were non-comparative [58-60] and one used cross sectional study design [61]. Five national level PD cohort studies [48,51,52,55,56] used information from the French Language Peritoneal Dialysis Registry (RDPLF). Two of these RDPLF studies [51,52] used the same time period but applied different exclusion criteria based on the focus of the studies*.* Two PD cohort studies [53,54] relied on data from regional dialysis units. The remaining three PD cohort studies [49,50,57] and all of the non-comparative and cross sectional PD studies [58-61] used data from a single hospital/dialysis unit. Of the 14 PD studies, 6 were from France [48,50-52,55,56], 3 from Canada [53,54,58], 2 from the United States of America (USA) [60,61] and one each from China [57], Taiwan [49] and Brazil [59].

**Table 1:** Overview of study characteristics

**Study**	**Type of study**	**Setting, data source & data period**	**Country**
***Peritoneal dialysis***			
Brunier et al. [58]	Non-Comparative (Case Series)	• Hospital	Canada
		• Sunnybrook Health Science Centre (November 1993 - May 1995)	
Castrale et al. [48]	Retrospective Cohort	• National	France
		• French Peritoneal Dialysis Registry Data (January 2000 - December 2007)	
Franco et al. [59]	Non-Comparative (Case Series)	• Clinic	Brazil
		• GAMEN Renal Clinic (January 2003- July 2009)	
Hsieh et al. [49]	Prospective Cohort	• Hospital	Taiwan
		• Chang Gung Memorial (January 2000 - December 2009)	
Lobbedez et al. [50]	Retrospective Cohort	• Hospital	France
		• Academic Hospital of Basse-Normandie (1 January 1998–31 December 2003)	
Lobbedez et al. [51]	Retrospective Cohort	• National	France
		• French Peritoneal Dialysis Registry Data (1 January 2002 – 1 June 2011)	
Lobbedez et al. [52]	Retrospective Cohort	• National	France
		• French Peritoneal Dialysis Registry Data (1 January 2002 – 1 June 2011)	
Oliver et al. [53]	Prospective Cohort	• Regional Dialysis Center	Canada
		• Sunnybrook Health Science Centre (1 January 2004–25 May 2006)	
Oliver et al. [54]	Prospective Cohort	• 4 Regional Dialysis Centers	Canada
		• Sunnybrook Health Science Centre (HSC), Halton Healthcare, London HSC, Manitoba Renal Program (January 2004 - January 2009)	
Ponferrada et al. [61]	Cross Sectional (Survey)	• Dialysis Unit	USA
		• Dialysis Clinic Inc (Data period not reported)	
Verger et al. [55]	Retrospective Cohort	• National	France
		• French Peritoneal Dialysis Registry Data (1 January 1995–1 January 2006)	
Verger et al. [56]	Retrospective Cohort	• National	France
		• French Peritoneal Dialysis Registry Data (1 January 2000 – 1 January 2005)	
Wadhwa et al. [60]	Non-Comparative (Case Series)	• Hospital	USA
		• Division of Nephrology and Hypertension - State University of New York (January 1989 - December 1992)	
Xu et al. [57]	Prospective Cohort	• Hospital	China
		• Peking University First Hospital (July 2002 - April 2010)	
***Hemodialysis***			
Agraharkar et al. [63]	Non-Comparative (Case Series)	• Citywide	USA
		• Dialysis centers in the greater Houston area (1995–1998)	
Babamohammadi et al. [62]	Randomized controlled trial	• Hospital	Iran
		• Fatemyeh Hospital (Data period not reported)	
***Pre-dialysis & dialysis***			
Wilde et al. [64]	Cross Sectional (Survey)	• Hospital	UK
		• Leicester General Hospital (Data period not reported)	

**Table 2:** Patient population, intervention type and major findings of studies

**Study**	**Number of patients**	**Mean age**	**Type of home care intervention**	**Major findings**
***Peritoneal dialysis***				
Brunier et al. [58]	• 18 HC assisted PD^α^	• 61	• HC^β^ (Nurse) assisted PD: Publicly funded program where nurses visit homes for PD exchanges and clinical and social support	*Peritonitis Rate:*
				• One episode of peritonitis per 33.8 patients-months (excluding 1 low white blood and 1 AIDS patient); if included, one episode of peritonitis per 20.1 patients-months
			• CAPD^γ^ required 1–4 visits / day based on severity of disability	*Hospitalization Rate:*
				• One hospital admission per 15.3 patient-months (excluding 5 palliative care patients)
			• CCPD^δ^ required 2 visits / day	*Costs:*
			• Reporting on 3 years of experience	• Annual costs were $27,263 for home CAPD, $29,763 for home CCPD and $29,915 for HD^ϵ^
Castrale et al. [48]	• 1232 HC assisted PD	• 83 (HC assisted PD)	• HC (Nurse) assisted PD: Publicly funded home visits by private sector nurses for assisted PD	*Peritonitis Rate:*
				• Compared to self care PD, HC (nurse) assisted PD had similar risks of peritonitis rates (Bivariate results)
	• 87 Family assisted PD	• 81 (Family assisted PD)	• Study end point allowed for at least 2 years of follow-up for patients who are on PD continuously (Frequency of visits not reported)	*Patient Survival:*
				• Compared to self care PD, HC (nurse) assisted PD was associated with a higher risk of mortality (RH^ζ^ = 2.35)
	• 294 Self care PD	• 80 (Self care PD)		*Technique Survival:*
		* Elderly		• Compared to self care PD, HC (nurse) assisted PD had similar risks of technique failure (=transfer to HD)
Franco et al. [59]	• 30 HC assisted PD	• 72 (Median)	• HC (Nurse Assistant) assisted PD: Home visits by nurse assistants for assisted PD	*Peritonitis Rate:*
				• One episode of peritonitis per 37 patient-months
			• Study end point allowed for at least 16 months of follow-up for patients who are on PD continuously (Frequency of visits not reported)	*Patient Survival:*
				• Patient survival was 60% at 12 months, 23% at 24 month, 3% at 48 months
			• Each nurse assistant was responsible for 4 patients	
Hsieh et al. [49]	• 32 HC assisted PD	• 76 (HC assisted PD)	• HC (Home Assistant) assisted PD: Home assistants (a background in healthcare was not required) paid by the family assisted with PD.	*Peritonitis Rate:*
				• Peritonitis rates of 1 episode per 24 (HC-home assistant-assisted PD), 37 (family assisted PD) and 39 (self PD) patient months were not significantly different
	• 44 Family assisted PD	• 74 (Family assisted PD)	• HC assisted PD patients followed for 93 to 1832 days (Frequency of visits not reported)	• Probability of a 12 month peritonitis-free periods of 62.5% (HC-home assistant-assisted PD), 75.0% (family assisted PD) and 80.8% (self PD) were not significantly different
	• 26 Self care PD	• 69 (Self care PD)		*Patient Survival:*
		* Elderly		• Peritonitis-related deaths of 13.8% (HC-home assistant-assisted PD), 8.0% (family assisted PD) and 8.0% (self PD) were not significantly different
				*Technique Survival:*
				• Following peritonitis, technique failures of 34.5% (HC-home assistant-assisted PD), 16.0% (family assisted PD) and 16.0% (self PD) were not significantly different
Lobbedez et al. [50]	• 36 HC assisted PD	• 74 (HC assisted PD)	• HC (Nurse) assisted PD: Publicly funded home visits by private sector nurses for assisted PD	*PD Eligibility/Utilization/Uptake:*
				• HC (nurse) assisted PD enables increased use of PD in incident dialysis patients
	• 61 Self care PD	• 52 (Self care PD)	• HC assisted PD patients followed for 0.5 to 51 months (Frequency of visits not reported)	*Peritonitis Rate:*
				• HC (nurse) assisted PD patients: Actuarial survival free of peritonitis was 72% at 6 months, 50% at 12 months
	• 36 Satellite HD	• 47 (Satellite HD)		*Hospitalization Rate:*
	• 106 In-center HD	• 67 (In-center HD)		• HC (nurse) assisted PD patients:
				• Actuarial survival free of hospitalization 46% at 6 months, 21% at 12 months
				• Hospitalization rate was 0.4 admissions/patient/month
				*Technique Survival:*
				• HC (nurse) assisted PD patients: Technique survival 85% at 6 months, 58% at 12 months
				*Charlson Comorbidity Index:*
				• Charlson Comorbidity Index higher for HC (nurse) assisted PD (7.0) when compared to self care PD (4.3) and similar when compared to in-center HD (7.7)
Lobbedez et al. [51]	• 4230 HC assisted PD	• 79 (Median, HC assisted PD)	• HC (Nurse) assisted PD: Publicly funded home visits by private sector nurses for assisted PD	*Patient Survival:*
				• Compared to self care PD, HC (nurse) assisted PD was associated with a higher risk of mortality (cs-RH^η^ = 4.52)
	• 1056 Family PD	• 74 (Median, Family assisted PD)	• Study end point allowed for at least 5 months of follow-up for patients who are on PD continuously (Frequency of visits not reported)	• Compared to self care PD + family assisted PD, HC (nurse) assisted PD was associated with a higher risk of mortality (cs-RH = 2.18)
	• 4515 Self care PD	• 56 (Median, Self care PD)	• Interquartile range of PD duration 7.78 to 29.70 months	*Technique Survival:*
				• Compared to self care PD, HC (nurse) assisted PD was associated with a lower risk of technique failure (=transfer to HD, cs-RH = 0.84, sd-RH^θ^ = 0.72)
				• Compared to self care PD + family assisted PD, HC (nurse) assisted PD was associated with a lower risk of technique failure (=transfer to HD, cs-RH = 0.85, sd-RH = 0.72)
				*Renal Transplantation:*
				• Compared to self care PD, HC (nurse) assisted PD was associated with a lower risk of renal transplantation (cs-RH = 0.04)
				• Compared to self care PD + family assisted PD, HC (nurse) assisted PD was associated with a lower risk of renal transplantation (cs-RH = 0.16)
				*Renal Recovery:*
				• Compared to self care PD, HC (nurse) assisted PD was associated with a similar risk of renal recovery (Bivariate results)
				• Compared to self care PD + family assisted PD, HC (nurse) assisted PD was associated with a similar risk of renal recovery
Lobbedez et al. [52]	• 3689 HC assisted PD	• 71 (Median, Planned PD Start)	• HC (Nurse) assisted PD: Publicly funded home visits by private sector nurses for assisted PD	*Peritonitis Rate:*
				• Compared to self care PD, HC (nurse) assisted PD was associated with a lower risk of peritonitis (sd-RH = 0.81)
	• 902 Family PD	• 69 (Median, Sub-optimal PD Start)	• Study end point allowed for at least 5 months of follow-up for patients who are on PD continuously (Frequency of visits not reported)	*Patient Survival:*
				• Compared to self care PD, HC (nurse) assisted PD was associated with a higher risk of mortality (sd-RH = 6.30)
	• 3891 Self care PD		• Interquartile range of PD duration 8.08 to 29.99 months	*Technique Survival:*
				• Compared to self care PD, HC (nurse) assisted PD was associated with a lower risk of technique failure (=transfer to HD, sd-RH = 0.67)
	*Additional exclusions compared o Lobbedez et al., 2012 to focus on sub-optimal PD starts			*Renal Transplantation:*
				• Compared to self care PD, HC (nurse) assisted PD was associated with a lower risk of renal transplantation (sd-RH = 0.03) (Bivariate results)
Oliver et al. [53]	• 22 HC assisted PD	• 76 (Median, HC assisted PD)	• HC (Nurse) assisted PD: Publicly funded program where nurses visit homes for PD exchanges and clinical and social support	*PD Eligibility/Utilization/Uptake:*
				• More elderly patients were considered eligible for PD (OR^ι^ = 2.6) if they lived in a HC support region than if they did not
	• 4 Self care PD	• 76 (Median, Other Modalities)	• First year of dialysis, HC assisted PD patients received, on average, 5.8 visits / week	*Hospitalization Rate & Days:*
				• Hospitalization rate was not significantly different between HC (nurse) assisted PD (1.4 per patient-year) and other modalities (1.0 per patient-year)
	• 16 In-center HD	*Elderly	• HC assisted PD patients offered 2 visits / day 7 days a week	• Hospital days were not significantly different between HC (nurse) assisted PD (23.5 per patient-year) and other modalities (13.1 per patient-year)
			• Mean follow-up for HC assisted PD patients were 413 days	*Patient Survival:*
				• Mortality was not significantly different between HC (nurse) assisted PD (0.12 per patient-year) and other modalities (0.18 per patient-year)
				*Technique Survival:*
				• Modality changes were not significantly different between HC (nurse) assisted PD (0.04 per patient-year) and other modalities (0.19 per patient-year)
Oliver et al. [54]	• 56 HC assisted PD	• 66 (Overall)	• HC (Nurse or Healthcare Aid) assisted PD: Publicly funded program where nurses or healthcare aids visit homes for assisted PD	*PD Eligibility/Utilization/Uptake:*
				• Even when HC (nurse or healthcare aid) assisted PD is available, family support remains to be an important driver of PD utilization.
	• 8 Family and HC assisted PD		• Maximum 2 nurse or healthcare aid visits/day	• Among patients with barriers to PD who live in areas with HC assisted PD availability, PD utilization was higher (39%) among those who had family support compared to those without family support (23%)
	• 26 Family assisted PD		• Mean (median) follow-up for PD patients were 521 (376) days	
	• 1 Friend assisted PD			
	• 56 Self care PD			
Ponferrada et al. [61]	• 36 HC assisted PD	• 55	• HC (Team) assisted PD: Home visits by home care team (nurse, dietician & social worker) for assisted PD and patient assessments	*Critical Elements of Home Visits:*
				• Dialysis programs should retain the option of making home visits to home dialysis patients
			• Reporting on evaluation over a 18-month period	• To evaluate internal policy and identify critical elements of a home visit
			• Study recommendations: One routine visit for new patients and additional non-routine visits only if there are significant problems	
			• A visit took approximately 4 hours	
Verger et al. [55]	• 5284 HC assisted PD	• 66 (Overall)	• HC (Nurse) assisted PD: Publicly funded home visits by private sector nurses for assisted PD	*PD Eligibility/Utilization/Uptake:*
				• Provides a description of the PD population
	• 822 Family assisted PD		• Nurse time at patient’s home for: a) non-disconnect CAPD ultraviolet system between 10–15 minutes, and b) double-bag disconnect CAPD system between 30–45 minutes (Frequency of visits not reported)	• Over the decade studied, 45% of all incident PD patients received HC (nurse) assisted PD and 87% of incident PD patients over 90 years of age received HC (nurse) assisted PD
	• 8285 Self care PD			*Charlson Comorbidity Index:*
				• Among prevalent PD patients, Charlson comorbidity index, on average, was 7.6 for HC (nurse) assisted PD, 6.6 for family assisted PD and 4.8 for self care PD
	• 352 Other / Undefined PD			
Verger et al. [56]	• 232 HC assisted PD	• 73 (HC assisted PD)	• HC (Nurse) assisted PD: Publicly funded home visits by private sector nurses for assisted PD	*Peritonitis Rate:*
				• The probability of being peritonitis free at 24 months better for family assisted PD (76.7%) compared to HC (nurse) assisted PD (41.2%) when nurse visits from dialysis centers are not considered
	• 127 Family assisted PD	• 65 (Family assisted PD)	• Study end point allowed for at least 13 months of follow-up for patients who are on PD continuously	• The probability of being peritonitis free at 24 months similar between family assisted PD (57.7%) and HC (nurse) assisted PD (60.7%) when nurse visits from dialysis centers are considered
	• 1265 Self care PD	• 51 (Self care PD)	• 1–2 nurse visits / day	• For HC (nurse) assisted PD, the probability of being peritonitis free better for those affiliated with dialysis centers with nurse visits
	*Automated PD patients only			• For family assisted PD, the probability of being peritonitis free similar across centers with and without nurse visits
Wadhwa et al. [60]	• 21 HC assisted PD	• 62	• HC (Nurse) assisted PD: Home visits by nurses for assisted PD and clinical support	*Peritonitis Rate:*
				• One episode of peritonitis per 13 patient-months
			• Mean number of nursing hours per day was 13 (Frequency of visits not reported)	*Hospitalization Rate & Days:*
				• One hospital admission per 6 patient-months
				• Mean hospitalization days of 9 per admission
Xu et al. [57]	• 36 HC assisted PD	• 71 (HC assisted PD)	• HC (Home Assistant) assisted PD: Home assistants (a healthcare background was not required) paid by the family assisted with PD	*Peritonitis Rate:*
				• First episode of peritonitis was not significantly different between HC (home assistant) assisted PD and family assisted PD
	• 86 Family assisted PD	• 66 (Family assisted PD)	• PD patients followed for 1 to 88 months (Frequency of visits not reported)	*Patient Survival:*
				• Compared to family assisted PD, HC (home assistant) assisted PD was associated with higher risk of mortality (HR = 2.14)
	• 191 Self care PD	• 55 (Self care PD)		*Technique Survival:*
				• Technique survival was not significantly different between HC (home assistant) assisted PD (69.8 months) and family assisted PD (74.8 months)
***Hemodialysis***				
Agraharkar et al. [63]	• 28 HC assisted HD	• 69	• HC (Nurse) Assisted HD: Home visit by registered nurse for dialysis and clinical support	*Hospital Days:*
				• Mean hospitalization days of 9.43+/−1.83
			• Nephrologists also made home visits	*Costs:*
			• HD patients followed for 2 to 71 weeks	• Weekly ongoing costs of HC(nurse) assisted HD were $1200, in-center HD with ambulance transportation were $2640 and in-hospital dialysis were $5241
			• Frequency of visits determined by the nephrologist upon patient’s discharge from the hospital	
Babamohammadi et al. [62]	• 19 HC assisted HD	• 56 (HC assisted HD)	• HC (Nurse) Assisted HD: Visits every week before HD schedule for clinical support and retraining	*19 Clinical Outcomes:*
				• 15 out of the 19 items studied improved for home care group (weight gain, nausea, vomiting, headache, bone pain, weakness and fatigue, and itching decreased and general condition and levels of BUN, creatinine, potassium and phosphorus of the blood improved significantly. Changes in the mean values of blood pressure, pulse, temperature, sodium and calcium and hematocrit were not significant)
	• 18 HD without HC	• 58 (HD without HC)	• Mean follow-up for HC assisted HD patients were 27.1 months	
			• 4 visits / month	
***Pre-dialysis & Dialysis***				
Wilde et al. [64]	• 57 HC assisted PD or HD	• Not described	• HC (Team): Home care team (3 nurses & 1 renal care assistant) visits during pre-dialysis and dialysis phase of care for PD and HD patients	*Satisfaction with Home Care:*
				• Overall satisfaction with home care program: a) pre-dialysis phase of care − 76% very satisfied, 20% partly satisfied and b) dialysis phase of care − 80% very satisfied, 20% partly satisfied
			• Visits until transplantation, switch to hospital-based dialysis or death (Frequency of visits not reported)	

^*α*^*PD: Peritoneal Dialysis.*

^*β*^*HC: Home Care.*

^*γ*^*CAPD: Continuous Ambulatory Peritoneal Dialysis.*

^*δ*^*CCPD: Continuous Cycling Peritoneal Dialysis.*

^*ϵ*^*HD: Hemodialysis.*

*Elderly: Identifies studies focusing on patients at least 65 years of age and older.

^*ζ*^*RH: Relative Hazard.*

^*η*^*cs-HR: Cause-Specific RH.*

^*θ*^*sd-RH: Fine and Gray Sub-Distribution RH.*

^*ι*^*OR: Odds ratio.*

Of the 17 studies included in this study, 2 studies examined the impact HC has on HD patients. One of these studies was a randomized trial [62] conducted in an Iranian hospital and the second one was a non-comparative study that used information from citywide dialysis units in the USA [63].

In contrast to the general trend of studies included in this SR where the focus was exclusively on the dialysis phase of care for patients, one study [64] included information from both the dialysis and pre-dialysis phases of care for PD and HD patients. This study explored the impact HC has on patients in one hospital in the United Kingdom.

The studies included in this SR examined HC in a total of 15,058 patients (Table 2). Patients on PD treatment (with a total of 14,954 patients) constituted the dialysis population that was most frequently studied. While most studies focused on general dialysis populations, two PD studies [58,60] and one HD study [63] examined HC in special dialysis populations that had severe disability, terminal illness or complex comorbid conditions.

Of the 17 studies included in this review, 3 focused on elderly patients [48,49,53] with average age of the study samples ranging from 73 years [49] to 82 years [48]. In the remaining studies with pertinent information, average age ranged from 55 years [56,61] to 69 years [63]. The PD studies with comparators and pertinent information revealed that HC assisted PD patients had a higher average age ranging from 71 years [57] to 83 years [48] when compared to the overall age of study samples.

### Home care intervention

HC interventions primarily focused on the assistance provided during dialysis treatment (Table 2). The two studies that examined HC interventions for HD patients [62,63] and most of the HC assisted PD studies [48,50-53,55,56,58,60] focused on assistance dialysis patients received from a nurse. Two of the remaining studies [61,64] considered the effects of HC teams and the rest focused on assistance received from either a home-assistant where a background in healthcare was not necessary [49,57], a nurse assistant [59] or a nurse or a healthcare aid [54].

There are several factors, such as the severity of illness, the scope of HC provision and the requirements of dialysis technique used, that will influence the characteristics of a HC intervention. While patients on continuous cycling PD (CCPD) will mostly require two visits per day, those on continuous ambulatory PD (CAPD) may require one to four visits based on the severity of their disability [56,58]. The time that a HC worker spends at a CAPD patient’s home is dependent on the CAPD system used. PD exchange help for a patient on an ultraviolet non-disconnect CAPD system will usually require less time (about 10–15 minutes) compared to the time (about 30–45 minutes) needed for a patient on a double-bag disconnect CAPD system [55].

Based on studies with pertinent information, patients in Canada [53,54] were offered 14 visits per week for help with their PD exchanges and for the provision of clinical and social support. These patients received, on average, 5.8 visits per week during the first year of their dialysis [53]. In a USA program, a routine visit to a new PD patient was carried out to ensure proper installation of the cycler for an effective dialysis and non-routine visits were made only on an as-needed basis [61]. In this program, a visit took approximately four hours. Another USA program focused on ESRD patients with multiple medical and social problems [60]. In this program, a visit to help patients with their PD exchanges and to provide clinical and social support took, on average, 13 hours. In a HD study from Iran, the HC intervention was designed to conduct one visit per week before the HD schedule for clinical support and retraining [62].

### Outcomes

#### Peritoneal dialysis

While most of the PD studies focused on outcomes related to PD treatment (Table 2), a few provided insights into how the availability of assisted PD offers a choice to patients who are unable to perform their RRT independently. A description of the PD population in France [55], where healthcare system supports nurse assisted PD, revealed that 45% of all PD patients and 87% of those over 90 years of age were assisted by a nurse. Studies have shown that the availability of nurse assisted PD increases the eligibility for PD among elderly patients [53] and improved the uptake of PD in general [50]. One study [54] emphasized the importance of the availability of family assistance for PD utilization even in regions where HC assisted PD is available.

In PD studies with comparators, outcomes such as peritonitis rate and technique and patient survival constituted the main areas of focus. In general PD populations, studies using information from RDPLF concluded that technique failure/transfer to HD was lower among HC (nurse) assisted PD patients when compared to self care PD patients only [51,52] and to self care PD and family assisted PD patients as a group [51]. Another study in general PD populations [57], where home-assistants who were not required to have a background in healthcare helped PD patients, found that the probability of technique survival times were similar between HC assisted PD and family assisted PD patients. Studies that focused on elderly concluded that the probability of technique failure was similar between HC (nurse) assisted PD patients and comparators including patients on self care PD [48] and traditional modalities (i.e., self care PD and in-center HD) [53]. The probability of technique failure following an episode of peritonitis was also similar between home-assistant assisted PD and self care PD and family assisted PD patients [49].

Peritonitis rate was another outcome examined in PD studies with comparators. In almost all of these studies, HC assisted PD patients and the comparators (including family assisted PD among general PD populations [57], self care PD among elderly [48] and self care PD and family assisted PD among elderly [49]) had similar probabilities of being peritonitis free. In one study [52], HC assisted PD patients had lower peritonitis rates when compared to self care PD. In another study [56], HC assisted PD patients had higher peritonitis rates when compared to family assisted PD patients. However, the difference in peritonitis rates observed in the latter study disappeared when the effects of regular nurse visits from dialysis centers to the HC assisted PD patients were taken into consideration.

Most of the PD studies with comparators that examined patient survival found a higher probability of mortality among HC assisted PD patients. This result continued to hold across different comparators including self care PD among general PD populations [51,52], self care PD and family assisted PD among general PD populations [51], family assisted PD among general PD populations [57], and self care PD among elderly PD populations [48]. Two studies about elderly populations were exceptions. In the first study, the risk of mortality did not differ between patients receiving nurse assisted PD and those on traditional modalities [53]. In the second study, peritonitis-related mortality was similar among home-assistant assisted PD and self care PD and family assisted PD patients [49].

The PD studies without comparators focused on varying outcomes such as identifying critical elements of a home visit [61] and exploring costs of RRTs [58]. The studies that considered PD patients with severe disability reported peritonitis rates that ranged from 1 episode per 13.0 patient-months [60] to 20.1 patient-months [58] and hospitalization rates that ranged from 1 admission per 6.0 patient-months [60] to15.3 patient-months [58]. A recent study from Brazil [59] found 1 peritonitis episode per 37.0 patient-months and 60% patient survival at one year among a general PD population.

#### Hemodialysis

The HD study with a comparator [62] concluded that patients in HC group had improved on 15 of the 19 outcomes considered (including decreases in nausea, vomiting, headache, bone pain, weakness and fatigue and itching and improvements in general condition and the levels of creatinine, potassium and phosphorus of the blood). The HD study without a comparator [63] focused on patients diagnosed with terminal illness and found that, on average, patients were hospitalized for 9.43 days.

#### Pre-dialysis and dialysis

Based on survey results, more than three-fourths of PD and HD patients were very satisfied with the pre-dialysis and dialysis phase of their care after the implementation of HC program [64]. The HC team consisting of three nurses and one renal care assistant provided continuous social support to patients. The HC team also collected information about patients’ life goals and provided information to them about their dialysis modalities.

### Risk of bias and confounding

While most studies had low risk of bias in many domains (Table 3), apart from two studies [51,52] that used imputations techniques for missing information, loss to follow-up was rarely discussed. Some of the studies [48,49,51-53,56,57] have taken into consideration confounding variables such as age and Charlson Comorbidity Index (CCI). The CCI summarizes the impact comorbid conditions have on survival by assigning higher weights to more severe coexisting conditions such as metastatic carcinoma and lower weights to less severe ones such as dementia [65-67]. While CCI is one of the most widely used risk adjustment techniques in observational studies, the characteristics of CKD populations may require multidimensional prognostic indices that take into account physical, psychological, cognitive, functional and social factors [68]. One of the studies included in this SR reported on the physical performance of patients using Karnofsky Scale in addition to providing information on their comorbidity scores [59]. Apart from the descriptive information incorporated in the latter study, there were no studies that incorporated multidimensional indices as another confounding variable in their analysis.

**Table 3:** Risk of bias and confounding

**Risk of bias and confounding**		
**Q1: Do the inclusion/exclusion criteria remain identical across the comparison groups (the individuals) of the study?**		
Peritoneal Dialysis	Yes	Castrale [48], Franco [59], Hsieh [49], Lobbedez [51,52], Verger [55,56], Xu [57]
	Partially	Brunier [58], Lobbedez [50], Oliver [53,54], Ponferrada [61], Wadhwa [60]
Hemodialysis	Yes	Agraharkar [63], Babamohammadi [62]
Pre-dialysis & Dialysis	No	Wilde [64]
**Q2: Does the strategy for recruiting participants into the study remain identical across groups (individuals)?**		
Peritoneal Dialysis	Yes	Brunier [58], Castrale [48], Franco [59], Hsieh [49], Lobbedez [50-52], Oliver [53,54], Ponferrada [61], Verger [55,56], Wadhwa [60], Xu [57]
Hemodialysis	Yes	Agraharkar [63], Babamohammadi [62]
Pre-dialysis & Dialysis	Yes	Wilde [64]
**Q3: Is the selection of the comparison group appropriate, after taking into account feasibility and ethical considerations?**		
Peritoneal Dialysis	Yes	Castrale [48], Hsieh [49], Lobbedez [50-52], Oliver [53,54], Verger [55,56], Xu [57]
	Not Applicable	Brunier [58], Franco [59], Ponferrada [61], Wadhwa [60]
Hemodialysis	Yes	Babamohammadi [62]
	Not Applicable	Agraharkar [63]
Pre-dialysis & Dialysis	Not Applicable	Wilde [64]
**Q4: Does the study account for important variations in the execution of the study?**		
Peritoneal Dialysis	Yes	Castrale [48], Franco [59], Lobbedez [51,52], Oliver [53,54], Ponferrada [61], Verger [56]
	Partially	Brunier [58], Hsieh [49], Lobbedez [50], Wadhwa [60], Xu [57]
	Not Applicable	Verger [55]
Hemodialysis	Yes	Babamohammadi [62]
	Partially	Agraharkar [63]
Pre-dialysis & Dialysis	No	Wilde [64]
**Q5: Were valid and reliable measures, implemented consistently across all study participants used to assess inclusion/exclusion criteria, intervention/exposure outcomes, participant benefits and harms, and potential confounders?**		
Peritoneal Dialysis	Yes	Brunier [58], Castrale [48], Franco [59], Hsieh [49], Lobbedez [50-52], Oliver [53,54], Ponferrada [61], Verger [55,56], Wadhwa [60], Xu [57]
Hemodialysis	Yes	Agraharkar [63], Babamohammadi [62]
Pre-dialysis & Dialysis	Yes	Wilde [64]
**Q6: Was the length of follow-up identical across study groups or remedied through analysis?**		
Peritoneal Dialysis	Yes	Castrale [48], Lobbedez [51,52], Xu [57]
	No	Hsieh [49], Lobbedez [50], Oliver [53], Verger [56]
	Not Applicable	Brunier [58], Franco [59], Oliver [54], Ponferrada [61], Verger [55], Wadhwa [60]
Hemodialysis	Yes	Babamohammadi [62]
	Not Applicable	Agraharkar [63]
Pre-dialysis & Dialysis	Not Applicable	Wilde [64]
**Q7: In cases of high loss to follow-up (or differential loss to follow-up), was the impact assessed (e.g., through sensitivity analysis or other adjustment method)?**		
Peritoneal Dialysis	Yes	Lobbedez [51,52]
	No	Brunier [58], Oliver [53], Wadhwa [60]
	Not Applicable	Oliver [54], Ponferrada [61], Verger [55]
	Cannot Determine	Castrale [48], Franco [59], Hsieh [49], Lobbedez [50], Verger [56], Xu [57]
Hemodialysis	Cannot Determine	Agraharkar [63], Babamohammadi [62]
Pre-dialysis & Dialysis	No	Wilde [64]
**Q8: Are all important primary outcomes accounted for in the results?**		
Peritoneal Dialysis	Yes	Brunier [58], Castrale [48], Franco [59], Hsieh [49], Lobbedez [50-52], Oliver [53,54], Ponferrada [61], Verger [55,56], Wadhwa [60], Xu [57]
Hemodialysis	Yes	Babamohammadi [62]
	Partially	Agraharkar [63]
Pre-dialysis & Dialysis	Yes	Wilde [64]
**Q9: Are results believable taking study limitations into consideration?**		
Peritoneal Dialysis	Yes	Brunier [58], Castrale [48], Franco [59], Hsieh [49], Lobbedez [50-52], Oliver [53,54], Ponferrada [61], Verger [55,56], Wadhwa [60], Xu [57]
Hemodialysis	Yes	Agraharkar [63], Babamohammadi [62]
Pre-dialysis & Dialysis	Partially	Wilde [64]
**Q10: Did the study attempt to balance the allocation between the groups or match groups (e.g., through stratification, matching, propensity scores)?**		
Peritoneal Dialysis	Yes	Castrale [48], Lobbedez [51,52], Oliver [53], Verger [56], Xu [57]
	No	Hsieh [49], Lobbedez [50], Oliver [54], Verger [55]
	Not Applicable	Brunier [58], Franco [59], Ponferrada [61], Wadhwa [60]
Hemodialysis	Yes	Babamohammadi [62]
	Not Applicable	Agraharkar [63]
Pre-dialysis & Dialysis	Not Applicable	Wilde [64]
**Q11: Were important confounding variables taken into account in the design and/or analysis (e.g., through matching, stratification, interaction terms, multivariate analysis, or other statistical adjustment such as instrumental variables)?**		
Peritoneal Dialysis	Partially	Castrale [48], Franco [59], Hsieh [49], Lobbedez [51,52], Oliver [53], Verger [56], Xu [57]
	No	Brunier [58], Lobbedez [50], Oliver [54], Ponferrada [61], Verger [55], Wadhwa [60]
Hemodialysis	Yes	Babamohammadi [62]
	No	Agraharkar [63]
Pre-dialysis & Dialysis	No	Wilde [64]

## Discussion

Our SR revealed that most of the studies that examined the impact of HC services among CKD patients focused on dialysis patients, in general, and PD patients, in particular. Among RRTs, assisted PD provides an option for ESRD patients who prefer home-based dialysis therapies but have barriers to self care including physical disability and cognitive impairment. HC assisted PD becomes especially valuable for ESRD patients with additional barriers to self care such as social isolation. The current increases in the prevalence of elderly ESRD patients partly explain the greater emphasis placed on assisted PD in the CKD HC literature.

The HC assisted PD studies incorporated in this review mostly underscored clinically relevant outcomes for PD such as peritonitis rate and technique and patient survival. The findings show that technique survival and peritonitis rates for HC assisted PD patients were at least similar to or better than those for self care PD and family assisted PD patients. The studies that found better technique survival [51,52] and peritonitis rates [52] for HC (nurse) assisted PD patients relied on national level French registry using the same time period. The availability of HC assisted PD may reduce the likelihood of adverse events by improving patient’s psycho-social status and supporting them in adhering to the basic principles of PD including peritonitis prevention. Further studies are needed to examine if favorable outcomes continue to hold for HC assisted PD patients in different regions across the world.

Most PD studies found a higher probability of mortality among HC assisted PD patients when compared to self care PD or family assisted PD. These studies indicated that patients in their HC assisted PD group were older and had higher levels of comorbidities as captured by the CCI. The higher probability of mortality among HC assisted PD patients persisted in studies that controlled for age and CCI differences across groups. The authors mostly attributed this finding to data insufficiencies in capturing disabilities among PD populations. Apart from one study [59] that described comorbidity and physical performance in their study population, there were no studies that incorporated multidimensional indices that take into account physical, psychological, cognitive, functional and social factors as another confounding variable in their analysis.

The studies included in this SR provided limited information about the characteristics of the HC interventions. In general, technical requirements imposed on HC intervention based on the dialysis type used are well known among the CKD community. Additional studies that consider HC interventions with varying scope and frequency and duration of visits in different CKD populations will provide helpful information to the CKD community, especially for those who are considering HC programs for their own clinic/practice.

One of the gaps in the literature that was identified by our SR is related to the provision of HC services among non-ESRD CKD populations. Apart from one study [64] that incorporated information about patient experiences with the implementation of a HC program that affected both the dialysis and pre-dialysis phases of their care, there were no studies that explored the impact HC has on non-ESRD CKD populations. It is well known that CKD is often accompanied by several comorbid conditions, is common among older people and its prevalence increases with age. As emphasized by the World Kidney Day 2014 Steering Committee [69], these characteristics of CKD coupled with increased life expectancy worldwide call for further explorations into ways of optimizing health for elderly populations. The impact different HC services might have in improving health among non-ESRD CKD patients is one such area that deserves further explorations.

The lack of studies on the impact home palliative care has on patients with CKD is another gap in the literature that was identified by our SR. The quality of life considerations for CKD patients who are at the advance stages of their disease require focus on several issues including the management of their physical and psycho-social symptoms and the development of an advanced care plan that sets the goals for their care [70-74]. Studies that examine the impact home palliative care has on patients with CKD who are at the advance stages of their disease will help further advance the integration of palliative and renal care.

Our SR identified other gaps in the literature. There were no studies about HC provision among kidney transplant patients. Studies related to HC provision among HD patients were limited to small samples.

The HC interventions incorporated in the CKD literature were mostly limited to nursing care for ESRD patients. There were no studies about the provision of home support for activities of daily living or respite care for caregivers of CKD patients. While HC may become more important as CKD severity increases and, in the case of ESRD, it may be most useful for patients on home-based dialysis modalities or for those who choose conservative care, further studies are needed to quantify these differing levels of HC use and its impact.

One of the strengths of our study is the comprehensive SR conducted on a topic where there were, to the best of our knowledge, no previous SR undertaken. The comprehensive electronic database searches coupled with hand searches and expert consultations resulted in the identification of several gaps in the literature.

Our study has several limitations. One of the limitations of our study is arising from the subject matter itself. HC, as encompassing a diverse set of medical and psycho-social services, is one of the health services research areas that are constantly evolving with limited standardization in terminology. Our study which focused on the intersection of home care with CKD faced additional challenges given the changes in CKD definition itself in the past years that is continuing through today [75]. We made an attempt to balance this fundamental limitation by conducting comprehensive database searches, extensive hand searches and expert consultations. Second limitation of our study is the layered approached followed in study selection. Third limitation is the focus on studies published in English. Given the diversity of HC services, resource and time considerations were crucial factors in our decision to follow a layered study selection approach and to focus on studies published in English. As indicated above, we made an attempt to balance these limitations by conducting comprehensive database searches, extensive hand searches and expert consultations. Another limitation is our inability to conduct a meta-analysis for our study. The diversity of patient populations, HC interventions and outcomes studied made it impossible to conduct a meta-analysis.

## Conclusions

In this era of aging world population and medical and technical advances, chronic diseases, such as CKD, are growing in incidence and prevalence. HC may be useful in providing a more efficient and higher quality care for CKD patients. However, a synthesis of evidence on the effects of HC among CKD patients has not been undertaken. Our SR, which aimed at filling this void, revealed that extant studies almost exclusively focused on nurse assisted HC patients examining mostly PD related clinical outcomes. Our study concluded that there are several gaps in the literature. Specifically, there were no studies in areas such as home support for activities of daily living, palliative care at home or respite care for caregivers of CKD patients, in general, or for ESRD patients, in particular.

## Appendix

MEDLINE Search Strategy:

1. exp kidney diseases/

2. exp renal replacement therapy/

3. ((kidney or renal) adj2 (disease* or failure or damage or insufficiency)).mp.

4. ((kidney or renal) adj2 (transplant*5 or dysfunction or therap*)).mp.

5. (dialysis or dialyses or haemodialysis).mp.

6. Kidney, Artificial/

7. (kidney* adj artificial).mp.

8. or/1-7 (540012)

9. exp home care services/

10. (domiciliary adj3 (care or service$ or nurs$)).mp.

11. “home nurs$3”.mp.

12. ((home or care) adj3 (nonprofession$ or non-professional$)).mp.

13. (homemaker adj3 service$).mp.

14. (home adj3 service$).mp.

15. “home care”.mp.

16. (home adj3 (renal or dialys$3 or hemodialy$3 or peritoneal)).mp.

17. ((parenteral or nutrition or feeding) adj home).mp.

18. (“hospital at home” or “hospital in the home” or “in-home care”).mp.

19. “home health care”.mp.

20. (Home adj3 (rehabilitation or occupational or physical or physiotherap$ or social worker$ or speech)).mp.

21. Day Care/

22. (palliative adj5 home).mp.

23. ((caregiver$ or care-giver$ or carer$) adj3 “respite care”).mp.

24. ((long-term or long term) adj3 (home care or home-care)).mp.

25. activities of daily living.sh. and home.tw.

26. (personal care adj3 home).mp.

27. (self-care adj3 home).mp.

28. (day adj3 care).mp.

29. self care.sh. and home.tw.

30. or/9-29

31. 8 and 30

32. limit 31 to (english language and yr=“1990 -Current”)

33. limit 32 to “all child (0 to 18 years)”

34. 32 not 33.

## Abbreviations

CKD: Chronic kidney disease; RRT: Renal replacement therapy; ESRD: End-stage renal disease; HC: Home care; PD: Peritoneal dialysis; HD: Hemodialysis; HHD: Home hemodialysis; SR: Systematic review; KDIGO: Kidney Disease: Improving Global Outcomes; RDPLF: French Language Peritoneal Dialysis Registry; CCI: Charlson Comorbidity Index; CCPD: Continuous cycling PD; CAPD: Continuous ambulatory PD.

## Competing interests

The authors declare that they have no competing interests.

## Authors’ contributions

SKA contributed to the conception and design, analysis and interpretation of results, and drafted the manuscript. PK contributed to the conception and design, interpretation of results and provided input for manuscript revision. OD contributed to the interpretation of results and provided input for manuscript revision. AL contributed to the conception and design, interpretation of results and provided input for manuscript revision. All authors have read and approved the final manuscript.

## Pre-publication history

The pre-publication history for this paper can be accessed here:

http://www.biomedcentral.com/1471-2369/15/118/prepub
